# Supplementary material for: Ephrin-A5 potentiates netrin-1 axon guidance by enhancing Neogenin availability
Source: Sci Rep. 2019 Aug 19;9:12009. doi: 10.1038/s41598-019-48519-0 (PMC6700147; doi:10.1038/s41598-019-48519-0)
Supplement: Supplementary file 1 — Supplementary Figures [file 41598_2019_48519_MOESM1_ESM.pdf]

Supplementary figures for the manuscript:

Ephrin-A5 potentiates netrin-1 axon guidance by enhancing Neogenin availability.

L.-P. Croteau[1,2], T.-J. Kao[3,4], and A. Kania[1,2*].

[1]Institut de recherches cliniques de Montréal (IRCM), Montréal, QC, H2W 1R7, Canada
[2]Department of Anatomy and Cell Biology and Division of Experimental Medicine, McGill University, Montréal, QC, H3A 2B2, Canada
[3]Graduate Institute of Neural Regenerative Medicine, College of Medical Science and Technology, Taipei Medical University, Taipei 110, Taiwan
[4]Technology and Center for Neurotrauma and Neuroregeneration, Taipei Medical University, Taipei 110, Taiwan

[*] Correspondence: artur.kania@ircm.qc.ca

ORCIDS IDs:
AK: 0000-0002-5209-2520

Supplementary Figure 1

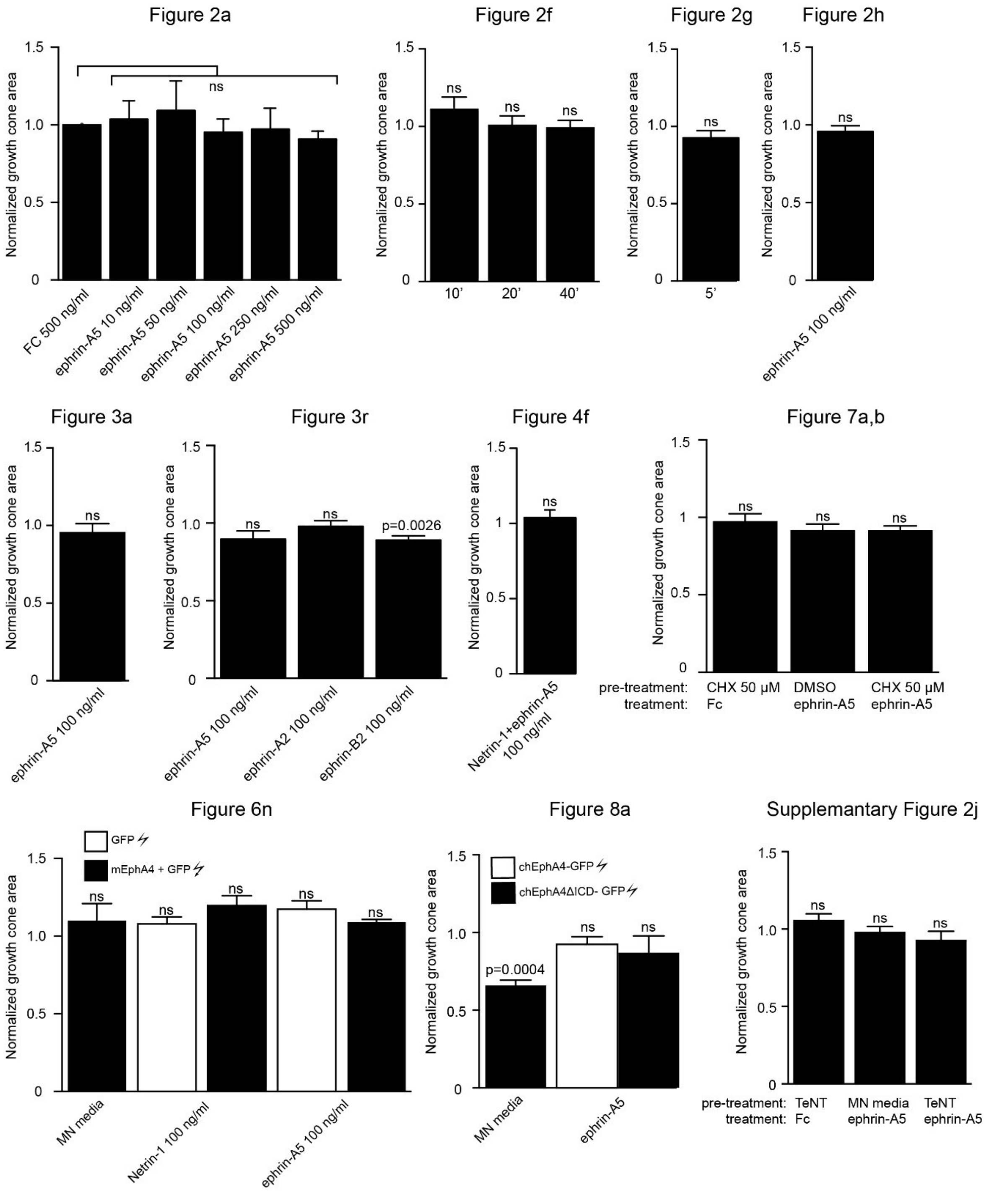

Mean area measurements of growth cones selected for IF analysis
(f) Compared to a 20' Fc 100 ng/mL treatment, ephrin-B2 100 ng/mL results in a 0.89-fold decrease in mean growth cone area (p=0.0026). (j) Compared to growth cones overexpressing EphA4-GFP, overexpression of EphA4ΔICD-GFP resulted in a 0.66-

fold decrease in mean growth cone area when treated with MN media for 20’ ($p=0.004$). In all other instances, mean growth cone measurements did not differ from controls. ($p>0.05$). Data are shown as mean ±SEM, statistical significance was tested using a two-tailed unpaired sample t-test.

Supplementary Figure 2

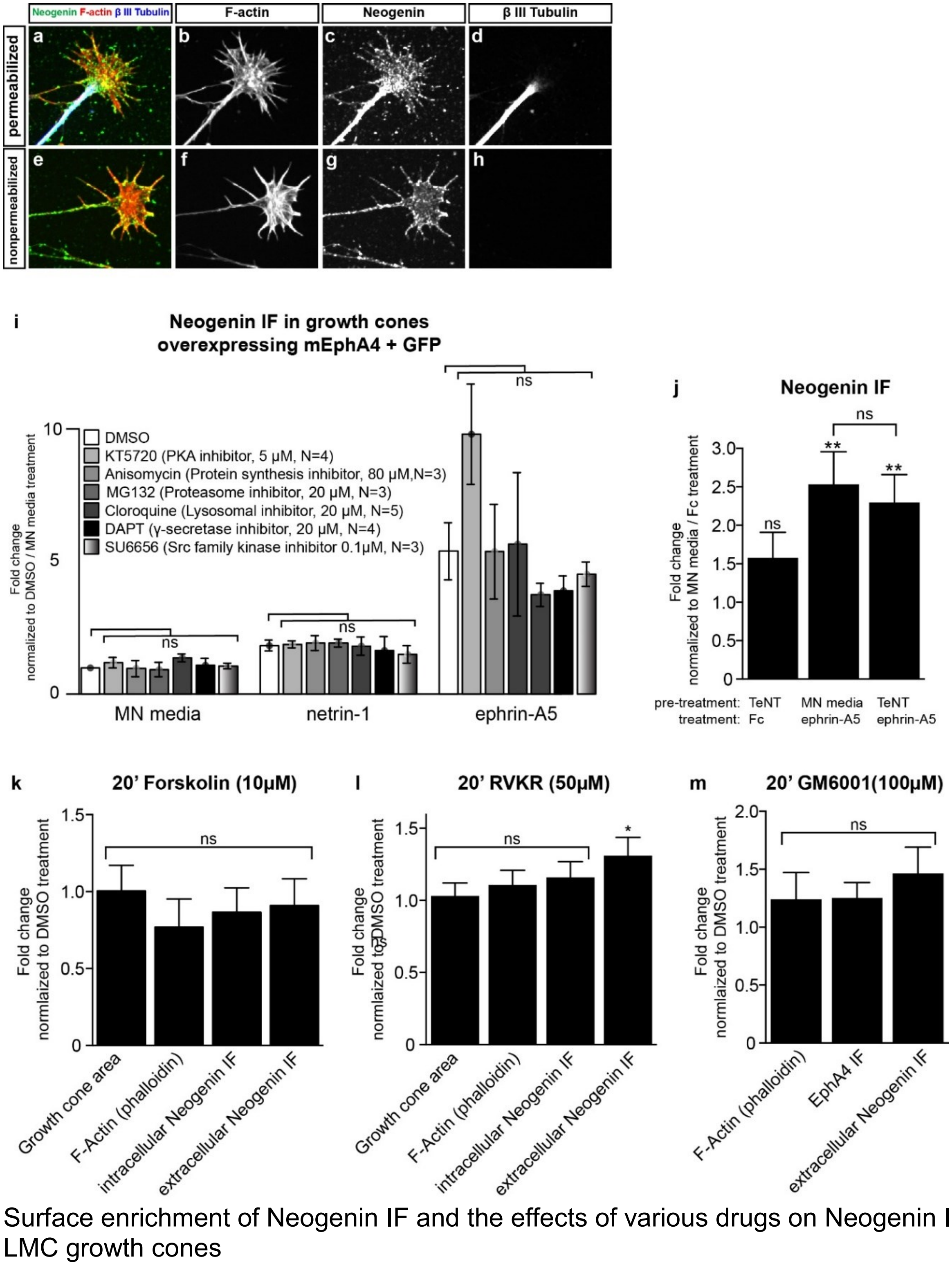

Surface enrichment of Neogenin IF and the effects of various drugs on Neogenin IF in LMC growth cones

(a-h) To confirm the surface enrichment of Neogenin when polyclonal anti-Neogenin antibody is added to live cultures (Figure 4a-c), an antibody against the intracellular protein βIII tubulin was included in fixed (a-d) and live (e-h) cultures, secondary antibodies and phalloidin to detect F-actin were added post-fixation (a-h). (i) The ephrin-A5 induced increase in Neogenin IF prevails despite inhibiting specific cell functions. LMC explants from chick spinal cords electroporated with a GFP expression plasmid in combination with a mEphA4 expression plasmid were incubated in the presence of various drugs for 20’ prior to a 20’ incubation with either MN media, netrin-1 or ephrin-A5 at 100 ng/mL followed by immunostaining for Neogenin. Drug treatments failed to modify Neogenin IF in GFP+ growth cones. (j) LMC explant cultures were pretreated for 20’ with tetanus toxin (TeNT, 2.87 nM) followed by a 10’ treatment with either Fc or ephrin-A5 (200 ng/mL). Relative to MN media / Fc treatment, MN media / ephrin-A5 and TeNT / ephrin-A5 treatments resulted in a 2.5 ± 0.4-fold ($p=0.004$) and a 2.3 ± 0.4-fold ($p=0.005$) increase in Neogenin IF respectively, pretreatment with TeNT did not significantly alter Neogenin IF in ephrin-A5 treated growth cones ($p=0.683$). (k-m) LMC explant cultures were treated for 20’ with either the adenylate cyclase activator Forskolin (j), the proprotein convertase inhibitor RVKR (k) or the metalloprotease inhibitor GM6001(l) followed by a quantification of F-actin, EphA4 and Neogenin IF in LMC growth cones. Relative to DMSO treatment, RVKR treatment results in 1.3 ± 0.1-fold increase Neogenin IF ($p=0.034$), all other measurements did not differ significantly ($p>0.05$). Data are shown as mean ±SEM, statistical significance was tested using a two-tailed unpaired sample t-test. (j) N=6 (k) N=4, (l) N=6, (m) N=5
